# Retinal vein changes in patients with high-risk proliferative diabetic retinopathy treated with conbercept and panretinal photocoagulation co-therapy: a cohort study

**DOI:** 10.3389/fendo.2023.1218442

**Published:** 2023-08-25

**Authors:** Mingwei Si, Yuan Tao, Ziniu Zhang, Hui Zhao, Wenxuan Cui, Mengyao Yang, Hong Wang

**Affiliations:** ^1^ Department of Ophthalmology, Qilu Hospital of Shandong University, Jinan, China; ^2^ Department of Ophthalmology, The Second People’s Hospital of Jinan, Jinan, China; ^3^ Department of Acupuncture and Moxibustion, Dongzhimen Hospital, Beijing University of Chinese Medicine, Beijing, China

**Keywords:** high-risk proliferative diabetic retinopathy, conbercept, panretinal photocoagulation, therapeutic effect, retinal vein diameter

## Abstract

**Objective:**

This study aimed to observe and compare retinal vein diameter changes and other essential indicators in patients with high-risk proliferative diabetic retinopathy (PDR) treated with intravitreal injection of conbercept (IVC) combined with panretinal photocoagulation (PRP) versus PRP monotherapy.

**Methods:**

A retrospective analysis was conducted on data from patients with high-risk PDR who received specific treatment and were followed up for 24 months. Among 82 patients with high-risk PDR, 50 eyes received PRP combined with IVC, whereas 32 eyes received PRP alone. During the 24-month follow-up period, changes in best-corrected visual acuity (BCVA), central foveal thickness (CFT), retinal vein diameter, number of microaneurysms (MA), neovascularization (NV) area, hard exudate (HE) area, size of the foveal avascular zone (FAZ), superficial capillary plexus (SCP) blood flow density, and adverse effects were recorded and compared between the two groups at baseline and at 6, 12, 18, and 24 months after treatment. The relationship between each observation index and vein diameter was also analyzed.

**Results:**

During the 24-month follow up, significant improvements in the BCVA, CFT, retinal vein diameter, number of MAs, NV area, HE area, FAZ, and SCP were observed in the IVC+PRP group after treatment. The PRP group only showed significant reductions in NV and HE areas. The IVC+PRP group showed significant superiority over the PRP group in improving the vein diameter, number of MA, and HE area. However, no statistically significant difference in NV area reduction was found between the groups.

**Conclusion:**

In the treatment of high-risk PDR, IVC+PRP therapy has a significant advantage over PRP monotherapy. IVC+PRP therapy may reverse diabetes-induced retinal vein changes, restoring morphology and function.

## 1 Introduction

Diabetic retinopathy (DR) is diabetes-induced microvascular damage to the retina, and it is currently the primary cause of avoidable blindness among the working-age population (1, 2). In 2020, 103.12 million people globally had DR, and it is projected to escalate to 160.50 million by 2045 (3). The duration of diabetes and severity of hyperglycemia are primary risk factors associated with DR (4). In addition, changes in retinal venous vessels can be observed in the early stages of diabetes, and a broader diameter of the retinal vein was identified as an independent risk factor for DR progression (5).

Proliferative DR (PDR) is an advanced stage of DR, and its diagnosis is based on the presence of retinal neovascularization (NV) or vitreous hemorrhage, which is caused by progressive damage to the retinal microvascular network (6, 7). PDR can be further defined based on the location and severity of NV, and a high-risk PDR is a more severe classification characterized by severe retinal ischemia and poor visual outcomes (8).

Panretinal photocoagulation (PRP) is still considered the conventional treatment for PDR (4). PRP involves destroying the non-perfused and ischemic areas of the peripheral retina, which results in retinal NV regression. This method aids in preserving central vision and decreasing the possibility of blindness in patients (6). The Early Treatment Diabetic Retinopathy Study (ETDRS) standard recommends early PRP in patients with high-risk PDR to effectively reduce the progression of retinal NV and PDR (9).

Anti-vascular endothelial growth factor (anti-VEGF) drugs, including ranibizumab, aflibercept, brolucizumab, faricima, conbercept, have been approved for the treatment of patients with DME, have changed the previous treatment paradigm of high-risk PDR with DME, which has demonstrated a crucial role in enhancing the best-corrected visual acuity (BCVA), lowering central foveal thickness (CFT), regulating exudation, and curbing NV (4, 10, 11). Conbercept is a recombinant soluble VEGF receptor decoy, like aflibercept. Both can fuse with the second immunoglobulin (Ig) domain of VEGF receptor 1 (VEGFR1), the third Ig domains of VEGFR2, the Fc region of human IgG and P1GF (12). Conbercept has an additional fourth Ig domain of VEGFR2 than aflibercept, which is critical for the receptor dimerization and the enhancement of the association rate of VEGF to the receptor (12). The SAILING study showed that using a pro re nata (PRN) IVC improved BCVA in patients with macular edema, and its efficacy was superior to laser photocoagulation therapy (13).

A meta-analysis reported that compared with PRP monotherapy, the combination therapy of IVC and PRP (IVC+PRP) had a more substantial effect on functional outcomes, such as bettering patient vision and decreasing macular edema (14). Moreover, previous retrospective studies have suggested the possibility of using anti-VEGF drugs to reverse DR by inducing changes in retinal pathology and physiology through reperfusion (15). Our previous study confirmed the efficacy of anti-VEGF drugs in patients with DR and observed a regression in vein diameter and venous beading after anti-VEGF therapy combined with PRP (16–18). However, no previous studies have focused on the changes in retinal vein diameter after IVC+PRP therapy. Current long-term research on the efficacy of IVC+PRP therapy in patients with high-risk PDR is limited, and changes in retinal pathology and physiology after anti-VEGF therapy and their significance need further exploration. Thus, in this retrospective cohort study, we aimed to compare and analyze the efficacy and changes in retinal vein diameter of the IVC+PRP group and PRP group in patients with high-risk PDR.

## 2 Materials and methods

### 2.1 Study design

This retrospective cohort study was conducted in compliance with the principles outlined in the Declaration of Helsinki. All patients gave informed consent for diagnosis and clinical procedures. Before treatment, all patients received detailed information about the potential risks involved, and they provided informed consent by signing a consent form for both IVC and PRP. The Medical Ethics Committee of Qilu Hospital of Shandong University approved this study (KYLL-2019-091).

### 2.2 Patients

Data of 82 patients with high-risk PDR (82 eyes) treated at Shandong University Qilu Hospital between January 2018 and December 2019 were retrospectively analyzed. In this study, high-risk PDR is defined based on the presence of NV accompanied by vitreous hemorrhage or NV without vitreous hemorrhage but occupying 1/3 to 1/4 of the optic disc area (8). Patients were divided into the IVC+PRP and PRP groups according to the treatment they received. The inclusion criteria were as follows: (1) patients with high-risk PDR diagnosed by fundus examination, fluorescein angiography (FFA), and optical coherence tomography (OCT); (2) patients with well-controlled blood glucose, glycated hemoglobin (GHb) <10%, and blood pressure <160/90 mmHg; (3) patients who received IVC+PRP or PRP monotherapy, and (4) patients followed up for a minimum of 24 months. The exclusion criteria were as follows: (1) Patients with diabetes types other than type 2 diabetes; (2) patients with other retinal diseases; (3) patients who underwent other types of intraocular therapies, including but not limited to intravitreal injection of other anti-VEGF agents or corticosteroids; and (4) patients with poor picture quality for various reasons including, but not limited to, refractive media opacities or proliferative membranes.

### 2.3 Data collection and follow up

The age, sex, eyes involved, disease duration, BCVA, CFT, retinal vein diameter, number of microaneurysms (MA), hard exudate (HE) area, NV area, size of the foveal avascular zone (FAZ), and superficial capillary plexus (SCP) blood flow density were collected by reviewing medical records. Adverse reactions during treatment were also recorded.

General information and medical history of patients were collected at the initial diagnosis. BCVA, color fundus photography (CFP), OCT, OCT angiography (OCTA), and FFA were performed on all patients at the initial diagnosis and at 6, 12, 18, and 24 months. The differences in each index before and after treatment and between groups at the above five time points were compared.

The primary efficacy analysis was based on changes in BCVA, CFT, and vein diameter. Other outcomes were considered secondary efficacy analyses.

BCVA was assessed using the ETDRS visual acuity chart. ZEISS Cirrus HD-OCT was used to acquire OCT images, and the built-in image processing software was used to measure CFT, which was the sum of subretinal fluid and neuroepithelial layer thickness. The Heidelberg Spectralis HRA fundus camera and video angiography were employed to measure the vein diameter, MA number, HE area, and NV area after image processing of the FFA. All vein measurements were taken from the photographs of the edge of 1–1.5 disc diameters centered on the optic disc. The maximum projected diameter of the largest six veins was manually measured, and the values were averaged to obtain the vein diameter using the proposed formula (19). For obtaining accurate and precise FFA results, images with a distinct outline of the retinal vein during the venous phase were used to eliminate the potential effect of neovascular leakage on the measurements. Following PRP, the examination of the peripheral retina was affected; therefore, the MA, HE area, and NV area were measured from FFA images of the retina within a 30-degree range centered on the fovea at 1 min. OCTA images were obtained using the ZEISS Cirrus HD-OCTA in the “HD Angio Retina 6 × 6 mm” mode, and FAZ and SCP were automatically measured. Two professional ophthalmologists reviewed all examination results.

In both groups, PRP was performed on patients by the same experienced ophthalmologist following the guidelines established by the DR Study Group (17). A frequency-doubled 532-nm laser (Lumenis Novus Omni, Lumenis Be, Inc., San Jose, USA) was used, with the patient’s eye fully dilated before treatment. The laser reaction was grade III, and the spot size was set to 200 μm, pulse width to 200 ms, and power to 230 mW. Laser therapy started from the posterior pole and extended to two disc diameters (PD) from the temporal side of the macula and superior and inferior vascular arcades, and one PD from the nasal side of the optic disc to the peripheral retina. The distance between laser spots was one spot diameter. PRP was performed in four sessions, with 300–400 laser spots applied in each session.

In the PRP group, the above treatment was employed at the initial diagnosis. If NV did not regress during follow up, additional laser therapy was performed.

The IVC+PRP group received an additional three + PRN regimen of the IVC based on the PRP mentioned above. All patients received IVC once a month for the first three months (0.05 mL/0.5 mg; Chengdu Kanghong Pharmaceutical Group Co., Ltd., China). All injections were performed by the same experienced physician.

The IVC+PRP group underwent the first PRP 1 week after the initial IVC injection, and if NV persisted or recurred after three injections, IVC+PRP therapy was repeated.

### 2.4 Statistical analysis

The IBM SPSS Statistics version 25.0 (IBM Corp., Armonk, NY, USA) was utilized to conduct the statistical analyses. Continuous variables are presented as means ± standard deviations, whereas categorical variables are presented as percentages (%). Paired t-tests were employed for normally distributed data, whereas non-parametric tests were employed for non-normally distributed ones. Count data were compared using chi-square tests. Statistical significance was established at *P* < 0.05. In the correlation analysis, |r| ≤ 0.3 indicated no linear correlation between variables.

## 3 Results

### 3.1 Baseline information

The baseline characteristics of the participants are shown in **Table 1**. This study included a total of 82 patients (82 eyes), of which 50 patients (50 eyes) were assigned to the IVC+PRP group and 32 patients (32 eyes) to the PRP group. The IVC+PRP and PRP groups did not exhibit any statistically significant differences in terms of age, sex, history of diabetes, number of MA, vein diameter, NV area, and HE area (*P* > 0.05; **Table 1**). However, significant differences were observed in BCVA and CFT (*P* < 0.01; **Table 1**).

**Table 1:** Baseline information.

	IVC+PRP (n = 50)	PRP (n = 32)	*t,χ2*	*P*
Sex, n (%)				
Male	26 (52.0%)	16 (50.0%)	0.03	0.86
Female	24 (48.0%)	16 (50.0%)		
Age (years)	62.12 ± 5.37	63.86 ± 6.03	1.37	0.18
Eyes				
Right eye	27 (54.0%)	15 (46.9%)	0.40	0.53
Left eye	23 (46.0%)	17 (53.1%)		
BCVA (LogMAR)	0.65 ± 0.15	0.46 ± 0.13	5.97	< 0.01
CFT (μm)	470.91 ± 44.14	295.43 ± 32.09	19.43	< 0.01
Duration of diabetes (years)	6.66 ± 2.14	7.58 ± 1.73	2.04	0.04

BCVA, best-corrected visual acuity; CFT, central foveal thickness.

### 3.2 BCVA and CFT

As significant differences in BCVA and CFT were found between the two groups at baseline, only BCVA and CFT changes in the IVC+PRP group were analyzed.

**Figure 1:**
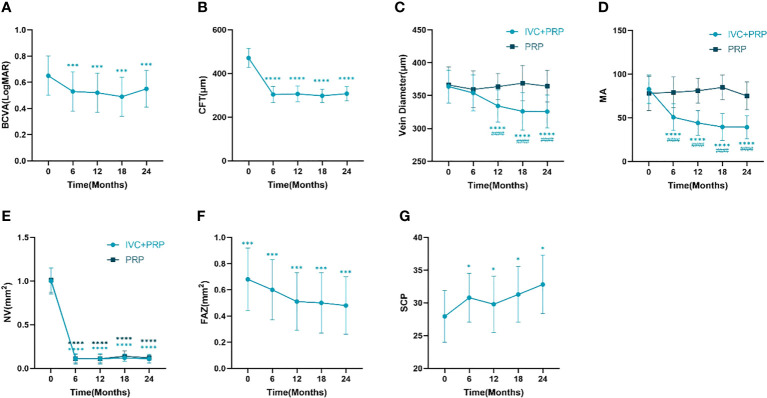
Changes in the BCVA (logMAR) **(A)**, CFT (mm) **(B)**, retinal vein diameter (mm) **(C)**, number of MA **(D)**, NV (mm^2^) **(E)**, FAZ (mm^2^) **(F)**, and vessel density of the SCP **(G)** at different time points between the IVC+PRP group and the PRP group. BCVA, best-corrected visual acuity. CFT, central foveal thickness. MA, microaneurysm. NV, neovascularization. FAZ, foveolar avascular zone. SCP, superficial retinal capillary plexus. *P < 0.05 *vs*. pretreatment, ***P < 0.001 *vs*. pretreatment, ****P < 0.0001 *vs*. pretreatment, ^####^ *P* < 0.0001 *vs*. PRP group.

The BCVA of the IVC+PRP group exhibited the superiority of the measurement at 6, 12, 18, and 24 months post-treatment over pretreatment measurement (*P* < 0.01, respectively, **Table 2**, **Figure 1A**). The CFT measurements at 6, 12, 18, and 24 months post-treatment were lower than pretreatment measurements (*P* < 0.01, respectively, **Table 2**, **Figure 1B**). BCVA and CFT improved significantly in the first 6 months, and the therapeutic effect was maintained in the subsequent treatment.

**Table 2:** Changes in BCVA (logMAR) and CFT (μm) at baseline *vs*. different time points post-treatment in the IVC+PRP group.

BCVA	Baseline	6 m	12 m	18 m	24 m	CFT	Baseline	6 m	12 m	18 m	24 m
n = 50	0.65 ± 0.15	0.53 ± 0.15	0.52 ± 0.13	0.49 ± 0.15	0.55 ± 0.14		470.91 ± 44.14	303.77 ± 37.54	306.62 ± 35.86	297.63 ± 30.71	307.35 ± 32.17
*t*		4.30	4.70	5.29	3.75			20.40	20.43	22.79	21.18
*P*		< 0.01	< 0.01	< 0.01	< 0.01			< 0.01	< 0.01	< 0.01	< 0.01

BCVA, best-corrected visual acuity; CFT, central foveal thickness; m, months.

### 3.3 Retinal vein diameter

Changes in the mean vein diameter in the two groups during the follow up are shown in **Table 3**, **Figure 1C**. At baseline, no significant difference was observed in the vein diameter between the two groups (*P* > 0.05). After IVC+PRP therapy, the retinal vein diameter in both groups was lower than that before treatment, and the diameter had a decreasing trend at 6 months of treatment, but without statistical significance (*P* > 0.05). At 12, 18, and 24 months of treatment, the decreasing trend of the retinal vein diameter continued at a slower rate, and the differences between the mean vein diameter and baseline were statistically significant (*P* < 0.01). No statistically significant difference in vein diameter changes was noted during the four follow up visits in the PRP group (*P* > 0.05). Compared with the PRP group, the IVC+PRP group showed significant vein diameter regression 12, 18, and 24 months after treatment (*P* < 0.01).

**Table 3:** Changes in vein diameter (μm) at baseline *vs*. different time points post-treatment between the IVC+PRP group and the PRP group.

	Baseline	6m	*t*	*P*	12 m	*t*	*P*	18 m	*t*	*P*	24 m	*t*	*P*
IVC+PRP (n = 50)	363.79 ± 24.95	354.01 ± 27.23	1.87	0.06	334.23 ± 24.71	5.95	< 0.01	326.05± 28.28	7.08	< 0.01	325.6 ± 25.16	7.61	< 0.01
PRP (n = 32)	366.11 ± 27.67	359.49± 27.89	0.95	0.34	363.81 ± 19.77	0.38	0.70	368.99± 26.71	0.42	0.67	364.34± 24.10	0.27	0.79
*t*	0.39	0.88			5.70			6.85			6.90		
*P*	0.70	0.38			< 0.01			< 0.01			< 0.01		

m, months.

### 3.4 MA, NV, and HE

Changes in MA, NV area, and HE area are shown in **Tables 4**–**6**, respectively.

**Table 4:** Changes in numbers of MA at baseline *vs*. different time points post-treatment between the IVC+PRP group and the PRP group.

	Baseline	6m	*t*	*P*	12 m	*t*	*P*	18 m	*t*	*P*	24 m	*t*	*P*
IVC+PRP (n = 50)	82.82 ± 16.47	50.66 ± 15.19	10.15	< 0.01	44.07 ± 14.29	12.57	< 0.01	39.50 ± 15.65	13.49	< 0.01	39.16 ± 13.39	14.54	< 0.01
PRP (n = 32)	78.04 ± 19.77	79.15 ± 17.68	0.24	0.81	80.95 ± 14.40	0.67	0.50	84.79 ± 14.10	1.57	0.12	75.09 ± 15.93	0.66	0.51
*t*	1.19	7.77			11.37			13.28			13.28		
*P*	0.24	< 0.01			< 0.01			< 0.01			< 0.01		

MA, microaneurysm; m, months.

**Table 5:** Changes in NV (mm^2^) at baseline *vs*. different time points post-treatment between the IVC+PRP group and the PRP group.

	Baseline	6 m	*t*	*P*	12 m	*t*	*P*	18 m	*t*	*P*	24 m	*t*	*P*
IVC+PRP (n = 50)	1.00 ± 0.15	0.11 ± 0.06	40.12	< 0.01	0.11 ± 0.06	40.34	< 0.01	0.12 ± 0.04	41.22	< 0.01	0.11 ± 0.05	40.54	< 0.01
PRP (n = 32)	1.01 ± 0.14	0.11 ± 0.05	35.25	< 0.01	0.11 ± 0.05	35.51	< 0.01	0.14 ± 0.06	33.25	< 0.01	0.12 ± 0.03	35.82	< 0.01
*t*	0.34	0.02			0.23			1.78			0.28		
*P*	0.73	0.98			0.82			0.08			0.78		

NV, neovascularization; m, months.

**Table 6:** Changes in the number of patients with different sizes of HE areas at baseline *vs*. different time points post-treatment between the IVC+PRP group and the PRP group.

		Baseline	6 m	*χ^2^*	*P*	12 m	*χ^2^*	*P*	18 m	*χ^2^*	*P*	24 m	*χ^2^*	*P*
IVC+PRP (n = 50)	No HE	1	2	2.73	0.43	4	7.81	0.05	5	13.79	< 0.01	9	18.88	< 0.01
	<0.5 mm^2^	11	15			19			22			21		
	0.5 mm^2^–2.5 mm^2^	20	22			19			18			16		
	>2.5 mm^2^	18	11			8			5			4		
PRP (n = 32)	No HE	1	1	0.34	0.95	1	3.36	0.34	2	5.59	0.13	2	6.31	0.10
	<0.5 mm^2^	7	9			12			14			15		
	0.5 mm^2^–2.5 mm^2^	12	11			13			11			10		
	>2.5 mm^2^	12	11			6			5			5		
*χ^2^*		0.14	1.63			0.88			0.84			3.15		
*P*		0.99	0.65			0.83			0.83			0.37		

HE, hard exudate; m, months.

For MA, the IVC+PRP group showed a decreasing trend throughout the treatment process, and the rate of decrease slowed down with the extension of treatment time (**Figure 1D**). The decrease in the number of MA at each follow-up visit was statistically significant when compared with the number before treatment (*P* < 0.01). The change in the PRP group was not significant (*P* > 0.05), and the difference between the two groups was significant (*P* < 0.01).

For the NV area, no statistically significant difference was found between the IVC+PRP and PRP groups at baseline (*P* > 0.05). Both groups showed a significant decrease in the NV area in the first 6 months of treatment, followed by a stable trend (**Figure 1E**). The NV area decreased significantly in both groups at each follow-up point after treatment (*P* < 0.01), and no significant difference was found between the two groups (*P* > 0.05).

For HE, both groups demonstrated a statistically significant reduction in the number of patients with different sizes of HE areas before and after treatment (*P* < 0.05). However, no significant statistical difference was observed between the two groups. The number of patients with no HE after treatment in the IVC+PRP group significantly increased, whereas in the PRP group, more patients had HE <0.5 mm^2^ after treatment than those with larger HE areas.

### 3.5 FAZ and SCP

Changes in the FAZ and SCP of the IVC+PRP group are shown in **Table 7** and **Figure 1**. Throughout treatment, the change in the FAZ showed a downward trend, with a significant downward trend in the first 12 months and a slowing trend from 12 to 24 months (**Figure 1F**). Compared with baseline, the FAZ was significantly reduced at 6, 12, 18, and 24 months of treatment (*P* < 0.01). Moreover, the SCP improved, with a rise at 6 months, a slight rebound at 12 months, and a continued upward trend in subsequent treatments (**Figure 1G**). Compared with baseline, the SCP also showed significant improvement at 6, 12, 18, and 24 months of treatment (*P* < 0.05).

**Table 7:** Changes in FAZ (mm^2^) and vessel density of SCP at baseline *vs*. different time points post-treatment in the IVC+PRP group.

FAZ	Baseline	6 m	12 m	18 m	24 m	SCP	Baseline	6 m	12 m	18 m	24 m
N = 50	0.68 ± 0.24	0.60 ± 0.23	0.51 ± 0.22	0.50 ± 0.23	0.48 ± 0.22		27.94 ± 3.95	30.79 ± 3.71	29.79 ± 4.31	31.29 ± 4.26	32.82 ± 4.46
*t*		1.57	3.61	3.66	4.23			3.7162	2.2356	4.0710	5.78
*P*		0.12	< 0.01	< 0.01	< 0.01			< 0.01	0.03	< 0.01	< 0.01

FAZ, foveolar avascular zone; SCP, superficial retinal capillary plexus; m, months.

### 3.6 Correlation coefficients

Correlation analysis was performed for the significant parameters in the IVC+PRP group before and after treatment. All values showed good statistical significance. Only a weak positive correlation was discovered between the vein diameter and CFT (r = 0.37215446, *P* < 0.01).

### 3.7 Adverse effects

No severe adverse effects were reported. Four patients in the combination group experienced subconjunctival hemorrhage after injection, which resolved spontaneously.

## 4 Discussion

This study aimed to observe the efficacy and changes in the retinal vein of patients with high-risk PDR treated with IVC+PRP and PRP therapy. To our knowledge, this is the first study to quantify changes in the retinal vein diameter after treatment with conbercept. Moreover, the combined use of conbercept and PRP exerted a significant effect on high-risk PDR, and this combination therapy may reverse the DR-induced changes in the retinal vein.

During the 2-year follow up, anti-VEGF combined with PRP for patients with high-risk PDR significantly improved the BCVA and reduced the CFT, which is consistent with previous reports (20). A study compared the efficacy of ranibizumab combined with PRP versus PRP alone in treating patients with high-risk PDR. The results showed that within 48 weeks, both groups had significantly reduced fluorescein angiography leakage; however, the combination group showed greater improvements in visual acuity, central macular thickness, and vascular leakage (21). In our previous retrospective study, we evaluated whether anti-VEGF treatment combined with PRP could reverse DR within a short treatment period. That study included 52 patients with high-risk PDR (72 eyes), which were divided into an aflibercept combination group and a PRP group. In the 6-month follow up, the combination group had significantly improved BCVA and CFT compared with the PRP group, with statistically significant differences in efficacy (17).

Improvement in the BCVA may be related to the degree of macular edema after treatment, and IVC+PRP therapy can alleviate macular edema and exudation by suppressing VEGF expression and improving vascular permeability, thereby improving macular morphology and function. The SAILING study and its extension study evaluated the safety and efficacy of aflibercept in patients with diabetic macular edema and observed the average change in BCVA in patients with diabetic macular edema from baseline to 24 months. The experimental group received aflibercept therapy and sham laser therapy at baseline, whereas the control group received sham injections or laser therapy at baseline, and both groups had repeated treatments based on monthly evaluations. The aflibercept therapy was more effective than traditional ETDRS laser photocoagulation therapy in improving vision and reducing CFT (13). In treating high-risk PDR, protecting the macula may be critical for improving BCVA.

Regarding changes in the retinal vein diameter, our results showed that in the 24-month follow up, IVC+PRP therapy effectively reduced the retinal vein diameter in patients with high-risk PDR, whereas no significant change was found in the vein diameter of the PRP group.

The change in the retinal vein diameter was consistent with our team’s previous observations in 59 patients with high-risk PDR (59 eyes) treated with 2-year aflibercept combined with PRP. After treatment, the BCVA and CFT significantly improved, and a statistically significant change in retinal vein diameter was found in the late stage of treatment. Notably, a regression of the vein bead was observed in the 18th month. Aflibercept intravitreal injection and PRP may reverse the retinal vein diameter and vein bead in the morphology of high-risk PDR (18).

A 12-month prospective cohort study reported that the retinal venous caliber decreased following treatment of diabetic macular edema with intravitreal anti-VEGF agents. Moreover, this decrease remained unchanged even when the injection regimen was switched from monthly treatment to a PRN regimen. Even in eyes that did not receive PRN aflibercept therapy exhibited persistent venous constriction at 12 months (22). The CLARITY study also observed changes in the retinal venous caliber and venous beading during treatment, and our results were consistent with their findings, demonstrating an improvement in BCVA, CFT, and intravitreal microvascular abnormalities at week 52 of aflibercept injection. Over 1 year of observation, the mean venous caliber and venous beading decreased; however, the decrease was not statistically significant (23). Fonseca believed that venous dilation and venous beading formation were chronic reactive dilations of the retinal veins to retinal ischemia, inflammatory response, or other abnormal conditions (24). We speculate that the retinal ischemia, hypoxia, and inflammation worsen with DR progression, causing the veins to reactively dilate to obtain more oxygen supply. Increased blood flow leads to an elevated venous hydrostatic pressure, and the vascular wall structure becomes fragile because of the apoptosis of vascular pericytes and thickening of the basement membrane caused by ischemia, hypoxia, and inflammation. The dilated venous vessels lose their elasticity and cannot recover, which may lead to a vicious cycle of further retinal vessel dilation caused by the burden of increased intravenous pressure. In the present study, the vein diameter of patients with PDR in the combined group changed significantly, and this may be due to the relief of retinal ischemia, leading to reductions in the hydrostatic pressure and pathological dilation of the vein diameter restored to a normal level. In addition, the anti-inflammatory effects of anti-VEGF drugs may also be a factor in vein diameter regression, new results showed the anti-inflammatory action of aflibercept in the retina damaged with high glucose *via* the PlGF/ERK pathway (25). Over time, retinal veins recovered their functions, and anatomical structure remodeling occurred. The results of the CLARITY study regarding changes in veins appear different from ours; however, our observation period was longer than that of the CLARITY study, and we found that venous changes appeared in the later stage of the 2-year treatment period. This may also indicate that anatomical changes in the retinal veins require sufficient time for venous remodeling.

Regarding secondary outcomes, we found that after 6 months of combined IVC and PRP, the NV area, number of MA, and HE area reduced in the treated eyes. The PRP group only showed improvement in the NV area.

Many studies have shown that both combination therapy and PRP monotherapy can eliminate NV. In our previous study on the use of aflibercept in combination with PRP to treat patients with high-risk PDR, the combination and PRP groups had statistically significant reductions in NV compared with baseline data during the 6-month observation period. However, no statistical difference was found between the two groups, and the combination group had a faster reduction in NV than the PRP group. Furthermore, we observed statistically significant improvements in BCVA, CFT, and MA in the combination group (17). Subgroup analysis from the CLARITY study revealed that during the 52-week observation period, 78.9% of the cases in the PRP group displayed partial regression of NV, with an average reduction in the NV area of 75.5% compared with the baseline. By contrast, all eyes in the anti-VEGF group exhibited complete regression of NV by week 12. Although both groups demonstrated significant NV regression, the combination group achieved earlier and more complete benefits (26). Sun also included two groups, i.e., IVC+PRP and PRP groups, followed up for 12 months. In addition, 70.88%, 29.12%, and 0% of eyes in the combination group had complete regression, partial regression, no regression, or NV progression, respectively, and in the PRP group, these were observed in 15.12%, 58.14%, 26.74% of eyes respectively, indicating better and earlier benefits in the combination therapy group (27). Preventing further vitreous hemorrhage and leakage is always our primary treatment goal in the prevention and treatment of NV. The efficacy of both IVC+PRP and PRP alone in improving NV regression was statistically significant after 24 months of treatment, indicating that both methods can achieve optimal efficacy by improving NV regression in patients with high-risk PDR. The combination therapy showed an earlier onset of efficacy compared with PRP monotherapy, which was also demonstrated in our previous study (17).

In our study, the combination group had reduced MA and HE area and was superior to the PRP group in all five observation points. Early studies have suggested that MA formation in patients with DR is caused by fragile vascular walls and vascular dilation, increased proliferation of endothelial cells, thickening of the basement membrane, and loss of pericytes (28). Animal experiments have shown that increased VEGF concentration in the vitreous body is related to MA formation (29), and the injection of anti-VEGF drugs can reduce VEGF concentration and other cytokines in the vitreous body (30). We speculate that MA and HE reductions in our study are caused by the loss of support for proliferating endothelial cells after the VEGF concentration decreased, which may lead to the cessation of proliferation or even apoptosis and ultimately result in DR regression. However, the influence of changes in the concentrations of other factors after injection cannot be excluded, and further experiments are needed to explore the relationship between each factor and MAs.

HE is primarily composed of dilated capillaries and lipids and proteins that leak into the interstitial space, mainly from MAs. The ETDRS reported that persistent central macular edema can progress to subretinal fibrosis with irreversible vision loss (31). A recent experiment suggested that HE can be an early predictive biomarker for HEs in DR (32). Previous studies have shown that HE significantly decreased after injections of anti-VEGF drugs (33), and our previous study also yielded similar results (17). In this study, the reduction in the HE in the combination group was superior to that in the PRP group. Therefore, because the injections of anti-VEGF drugs significantly reduce MAs, improve vascular permeability, and relatively restore lipid metabolism, HE is significantly reduced. The reduction in the HE area after anti-VEGF therapy may be related to MA reduction and the restoration of retinal vascular health after ischemia and hypoxia improved.

Quantitative indicators of FAZ and SCP in OCTA are related to the severity of DR (34–36). In this study, changes in the FAZ and SCP in the IVC+PRP group are consistent with previous research (37, 38), indicating that IVC+PRP therapy has a positive effect on macular perfusion status. These results suggest that IVC+PRP therapy can prevent the progression of DR-related non-perfusion.

Our trial is mainly limited by its retrospective design and a more restricted sample. Therefore, more prospective multicenter studies with larger sample sizes are warranted to comprehensively compare the effects of the two treatments. Our imaging results were all manually averaged over multiple measurements, which may introduce bias; thus, more professional equipment, targeted image processing software, and formulas are needed to provide more accurate results. Given the damage to the peripheral retina after PRP, we could not observe the condition of the peripheral retina. To monitor the treatment effect for a longer period, the first follow up was set at 6 months after treatment, which may not be suitable for observing the speed of onset between the two groups.

In summary, the combination therapy of IVC and PRP has advantages in BCVA improvement, CFT reduction, vein diameter restoration, reduction of the number of MAs, HE area, and FAZ, and improvement of SCP. Although the baseline BCVA and CFT of the PRP group are better than those of the IVC+PRP group, only the change in NV during treatment showed statistical significance compared with before treatment. The comparison between the two groups indicates the advantages of IVC+PRP therapy.

In the treatment of high-risk PDR, IVC+PRP therapy has a significant advantage over PRP monotherapy. Intravitreal injection of conbercept combined with PRP may reverse the retinal vein changes caused by diabetes in terms of morphology and function in DR.

## Data availability statement

The original contributions presented in the study are included in the article/supplementary material. Further inquiries can be directed to the corresponding author.

## Ethics statement

The studies involving human participants were reviewed and approved by The Medical Ethics Committee of Qilu Hospital of Shandong University. The patients/participants provided their written informed consent to participate in this study.

## Author contributions

MS, YT, and HZ contributed to conception and design of the study. MS wrote the manuscript. YT performed the statistical analysis. ZZ explained and visualized the data. WC and MY participated in the acquisition of data. HW performed the screening diagnosis and treatment of high-risk PDR. All authors contributed to manuscript revision, read, and approved the submitted version.

## References

[1] Cheung N Mitchell P Wong TY. Diabetic retinopathy. Lancet (2010) 376(9735):124–36. doi: 10.1016/S0140-6736(09)62124-3 20580421

[2] Chen L Cheng CY Choi H Ikram MK Sabanayagam C Tan GSW. Plasma metabonomic profiling of diabetic retinopathy. Diabetes (2016) 65(4):1099–108. doi: 10.2337/db15-0661 26822086

[3] Teo ZL Tham YC Yu M Chee ML Rim TH Cheung N. Global prevalence of diabetic retinopathy and projection of burden through 2045: systematic review and meta-analysis. Ophthalmology (2021) 128(11):1580–91. doi: 10.1016/j.ophtha.2021.04.027 33940045

[4] Martinez-Zapata MJ Salvador I Martí-Carvajal AJ Pijoan JI Cordero JA Ponomarev D. Anti-vascular endothelial growth factor for proliferative diabetic retinopathy. Cochrane Database Syst Rev (2023) 3(3):CD008721. doi: 10.1002/14651858.CD008721.pub2 36939655 PMC10026605

[5] Klein R Myers CE Lee KE Gangnon R Klein BEK. Changes in retinal vessel diameter and incidence and progression of diabetic retinopathy. Arch Ophthalmol (2012) 130(6):749–55. doi: 10.1001/archophthalmol.2011.2560 PMC3357449 22332203

[6] Moutray T Evans JR Lois N Armstrong DJ Peto T Azuara-Blanco A. Different lasers and techniques for proliferative diabetic retinopathy. Cochrane Database Systematic Rev (2018) 3(3):CD012314. doi: 10.1002/14651858.CD012314.pub2/full PMC6494342 29543992

[7] Lee R Wong TY Sabanayagam C. Epidemiology of diabetic retinopathy, diabetic macular edema and related vision loss. Eye Vis (Lond) (2015) 2:17. doi: 10.1186/s40662-015-0026-2 26605370 PMC4657234

[8] Flaxel CJ Adelman RA Bailey ST Fawzi A Lim JI Vemulakonda GA. Diabetic retinopathy preferred practice pattern. Ophthalmology (2020) 127(1):P66–145. doi: 10.1016/j.ophtha.2019.09.025 31757498

[9] The Diabetic Retinopathy Study Research Group. Indications for photocoagulation treatment of diabetic retinopathy: Diabetic Retinopathy Study Report no. 14. Int Ophthalmol Clin (1987) 27(4):239–53. doi: 10.1097/00004397-198702740-00004 2447027

[10] Sun JK Glassman AR Beaulieu WT Stockdale CR Bressler NM Flaxel C. Rationale and application of the protocol S anti-vascular endothelial growth factor algorithm for proliferative diabetic retinopathy. Ophthalmology (2019) 126(1):87–95. doi: 10.1016/j.ophtha.2018.08.001 30096354 PMC6916649

[11] Wang X He X Qi F Liu J Wu J. Different anti-vascular endothelial growth factor for patients with diabetic macular edema: A network meta-analysis. Front Pharmacol (2022) 13:876386. doi: 10.3389/fphar.2022.876386 35814207 PMC9260109

[12] Huang J Li X Li M Li S Xiao W Chen X. Effects of intravitreal injection of KH902, a vascular endothelial growth factor receptor decoy, on the retinas of streptozotocin-induced diabetic rats. Diabetes Obes Metab (2012) 14(7):644–53. doi: 10.1111/j.1463-1326.2012.01584.x 22340191

[13] Liu K Wang H He W Ye J Song Y Wang Y. Intravitreal conbercept for diabetic macular oedema: 2-year results from a randomised controlled trial and open-label extension study. Br J Ophthalmol (2022) 106(10):1436–43. doi: 10.1136/bjophthalmol-2020-318690 PMC9510409 34001667

[14] Huang C Ji H Han X. The effectiveness of conbercept combined with panretinal photocoagulation vs. Panretinal photocoagulation in the treatment of diabetic retinopathy: A meta-analysis. J Ophthalmol (2021) 2021:5591719. doi: 10.1155/2021/5591719 34046229 PMC8128542

[15] Levin AM Rusu I Orlin A Gupta MP Coombs P D’Amico DJ. Retinal reperfusion in diabetic retinopathy following treatment with anti-VEGF intravitreal injections. Clin Ophthalmol (2017) 11:193–200. doi: 10.2147/OPTH.S118807 28176934 PMC5268366

[16] Wang J Jiang PF Liu M Kou MR Lei JY Yu XT. Efficacy of intravitreal injection of conbercept on non-proliferative diabetic retinopathy: a retrospective study. J Int Med Res (2020) 48(4):300060519893176. doi: 10.1177/0300060519893176 32241206 PMC7132809

[17] Tao Y Jiang P Zhao Y Song L Ma Y Li Y. Retrospective study of aflibercept in combination therapy for high-risk proliferative diabetic retinopathy and diabetic maculopathy. Int Ophthalmol (2021) 41(6):2157–65. doi: 10.1007/s10792-021-01773-6 33772699

[18] Zhao H Wang J Li S Bao Y Zheng X Tao Y. Retinal vein changes after treatment with aflibercept and PRP in high-risk proliferative diabetic retinopathy. Front Med (2023) 10:1090964. doi: 10.3389/fmed.2023.1090964 PMC10034169 36968838

[19] Knudtson MD Lee KE Hubbard LD Wong TY Klein R Klein BEK. Revised formulas for summarizing retinal vessel diameters. Curr Eye Res (2003) 27(3):143–9. doi: 10.1076/ceyr.27.3.143.16049 14562179

[20] Zhang W Geng J Sang A. Effectiveness of panretinal photocoagulation plus intravitreal anti-VEGF treatment against PRP alone for diabetic retinopathy: A systematic review with meta-analysis. Front Endocrinol (Lausanne) (2022) 13:807687. doi: 10.3389/fendo.2022.807687 35422768 PMC9004461

[21] Filho JAR Messias A Almeida FPP Ribeiro JAS Costa RA Scott IU. Panretinal photocoagulation (PRP) versus PRP plus intravitreal ranibizumab for high-risk proliferative diabetic retinopathy. Acta Ophthalmol (2011) 89(7):e567–572. doi: 10.1111/j.1755-3768.2011.02184.x 21726427

[22] Blindbaek SL Peto T Grauslund J. Alterations in retinal arteriolar microvascular structure associate with higher treatment burden in patients with diabetic macular oedema: results from a 12-month prospective clinical trial. Acta Ophthalmol (2020) 98(4):353–9. doi: 10.1111/aos.14278 31654501

[23] Sivaprasad S Prevost AT Vasconcelos JC Riddell A Murphy C Kelly J. Clinical efficacy of intravitreal aflibercept versus panretinal photocoagulation for best corrected visual acuity in patients with proliferative diabetic retinopathy at 52 weeks (CLARITY): a multicentre, single-blinded, randomised, controlled, phase 2b, non-inferiority trial. Lancet (2017) 389(10085):2193–203. doi: 10.1016/S0140-6736(17)31193-5 28494920

[24] Fonseca RA Dantas MA. Retinal venous beading associated with recurrent branch vein occlusion. Can J Ophthalmol (2002) 37(3):182–3. doi: 10.1016/S0008-4182(02)80062-X 12083478

[25] Lazzara F Fidilio A Platania CBM Giurdanella G Salomone S Leggio GM. Aflibercept regulates retinal inflammation elicited by high glucose *via* the PlGF/ERK pathway. Biochem Pharmacol (2019) 168:341–51. doi: 10.1016/j.bcp.2019.07.021 31351870

[26] Nicholson L Crosby-Nwaobi R Vasconcelos JC Prevost AT Ramu J Riddell A. Mechanistic evaluation of panretinal photocoagulation versus aflibercept in proliferative diabetic retinopathy: CLARITY substudy. Invest Ophthalmol Vis Sci (2018) 59(10):4277–84. doi: 10.1167/iovs.17-23509 PMC6108778 30372756

[27] Sun Y Qi H. A comparison between the therapeutic effects of Conbercept combined with panretinal photocoagulation and panretinal photocoagulation monotherapy for high-risk proliferative diabetic retinopathy. Front Endocrinol (Lausanne) (2022) 13:1038757. doi: 10.3389/fendo.2022.1038757 36714571 PMC9880417

[28] Frank RN. Diabetic retinopathy. N Engl J Med (2004) 350(1):48–58. doi: 10.1056/NEJMra021678 14702427

[29] Tolentino MJ McLeod DS Taomoto M Otsuji T Adamis AP Lutty GA. Pathologic features of vascular endothelial growth factor-induced retinopathy in the nonhuman primate. Am J Ophthalmol (2002) 133(3):373–85. doi: 10.1016/S0002-9394(01)01381-2 11860975

[30] Mastropasqua R D’Aloisio R Di Nicola M Di Martino G Lamolinara A Di Antonio L. Relationship between aqueous humor cytokine level changes and retinal vascular changes after intravitreal aflibercept for diabetic macular edema. Sci Rep (2018) 8(1):16548. doi: 10.1038/s41598-018-35036-9 30410092 PMC6224583

[31] Fong DS Segal PP Myers F Ferris FL Hubbard LD Davis MD. Subretinal fibrosis in diabetic macular edema. ETDRS report 23. Early Treatment Diabetic Retinopathy Study Research Group. Arch Ophthalmol (1997) 115(7):873–7. doi: 10.1001/archopht.1997.01100160043006 9230827

[32] Shen Y Wang H Fang J Liu K Xu X. Novel insights into the mechanisms of hard exudate in diabetic retinopathy: Findings of serum lipidomic and metabolomics profiling. Heliyon (2023) 9(4):e15123. doi: 10.1016/j.heliyon.2023.e15123 37089301 PMC10119565

[33] Domalpally A Ip MS Ehrlich JS. Effects of intravitreal ranibizumab on retinal hard exudate in diabetic macular edema: findings from the RIDE and RISE phase III clinical trials. Ophthalmology (2015) 122(4):779–86. doi: 10.1016/j.ophtha.2014.10.028 25601535

[34] Sun Z Tang F Wong R Lok J Szeto SKH Chan JCK. OCT angiography metrics predict progression of diabetic retinopathy and development of diabetic macular edema: A prospective study. Ophthalmology (2019) 126(12):1675–84. doi: 10.1016/j.ophtha.2019.06.016 31358386

[35] Tang FY Chan EO Sun Z Wong R Lok J Szeto S. Clinically relevant factors associated with quantitative optical coherence tomography angiography metrics in deep capillary plexus in patients with diabetes. Eye Vis (Lond) (2020) 7:7. doi: 10.1186/s40662-019-0173-y 32025523 PMC6996172

[36] Lee H Lee M Chung H Kim HC. Quantification of retinal vessel tortuosity in diabetic retinopathy using optical coherence tomography angiography. Retina (2018) 38(5):976–85. doi: 10.1097/IAE.0000000000001618 28333883

[37] Lin W Feng M Liu T Wang Q Wang W Xie X. Microvascular changes after conbercept intravitreal injection of PDR with or without center-involved diabetic macular edema analyzed by OCTA. Front Med (Lausanne) (2022) 9:797087. doi: 10.3389/fmed.2022.797087 35391880 PMC8982760

[38] Zhu Z Liang Y Yan B Meng Z Long K Zhang Y. Clinical effect of conbercept on improving diabetic macular ischemia by OCT angiography. BMC Ophthalmol (2020) 20(1):382. doi: 10.1186/s12886-020-01648-x 32977791 PMC7519504

